# Supplementary material for: A laboratory perspective on accelerating preparatory processes before earthquakes and implications for foreshock detectability
Source: Nat Commun. 2024 Jul 3;15:5588. doi: 10.1038/s41467-024-49959-7 (PMC11222383; doi:10.1038/s41467-024-49959-7)
Supplement: Supplementary file 1 — Supplementary Information [file 41467_2024_49959_MOESM1_ESM.pdf]

Supplementary Material: A laboratory perspective on accelerating preparatory processes before earthquakes and implications for foreshock detectability

Thomas H. W. Goebel[a*], Valerian Schuster[b], Grzegorz Kwiatek[b], Kiran Pandey[a], and Georg Dresen[b]

[a]*University of Memphis, Center for Earthquake Research and Information, Memphis, Tennessee, USA.*
[b]*German Research Centre for Geosciences (GFZ), Section 4.2 Geomechanics and Scientific Drilling, Potsdam, Germany*
[*]corresponding author: thgoebel@memphis.edu

May 31, 2024

S1 Summary

This supplementary file presents more detailed information about sample geometry. loading conditions, bulk properties, system stiffness, focal mechanisms, fault micro-structure, crack density, sample dilation and seismic velocity changes. We start by reporting loading, boundary and initial conditions for each experiment (Tab. S1, Fig. S1). We then show sample bulk properties (Fig. S2), loading curves (Fig. S3), stiffness (Tab. S2, Fig. S4), focal mechanism examples (Fig. S5), microstructures (Fig. S6, S7), crack density (Fig. S8), pore volume and seismic velocity changes before rock fracture (Fig. S9, S10), average seismicity rates before slip (Fig. S11) and focal mechanism variability as a function of pore pressure (Fig. S12).

S2 Experimental conditions

Table S1: Summary table of experiments. P_c: confining pressure; **Roughness**: "Smooth" refers to cut and subsequently sanded surfaces with respective grid sizes in parentheses. "Rough" refers to freshly-fractured surfaces; **Notch**: length of each of the 30° inclined notches used to guide the fracture-process during fault creation, **Loading Rate**: far-field load-point velocity and **Pore pressure**: Servo-controlled pore fluid pressure within hydraulic system that was connected to the lab faults through a central borehole. Multiple values refer to different pore fluid pressures for subsequent stick-slip events. Note that here we focus on the first stick-slip events on fluid-saturated faults which are thought to be more similar in terms of initial surface roughness. The only exception is the dependence of shear type events on pore fluid pressure in Fig. 3c in the main text.

ID	P_c (MPa)	Roughness	Notch (mm)	Loading Rate (μm/sec)	Pore Pressure (MPa)	Thermal Damage
10	120	Smooth (#290)	0	0.3	dry	No
12	133	Smooth (#60)	0	0.3	dry	No
19	150	Rough	25	0.3	dry	No
20	150	Rough	25	0.3	0.5, 5	No
21	150	Rough	25	0.3	0.5, 5	Yes
22	150	Rough	25	0.3	15	Yes
23	150	Rough	25	0.3	5, 15	Yes
24	150	Rough	25	0.3	5, 25, 35	Yes
25	150	Rough	25	0.3	15	Yes
26	150	Rough	25	0.3	15	Yes

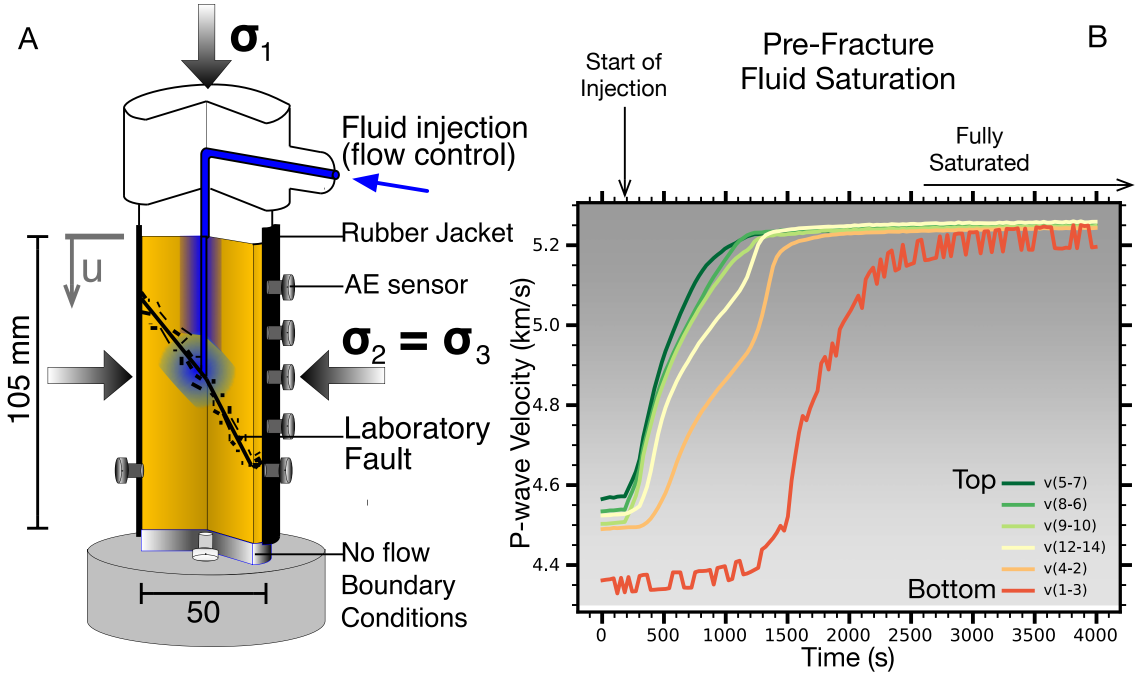

Figure S1: Left: Schematic illustration of sample geometry, loading conditions, AE sensor array and pore pressure system. Right: Seismic velocity changes during intact sample fluid-saturation through a central borehole drilled to the middle of the sample. Velocities increase systematically from top to bottom. Sample saturation takes about one hour.

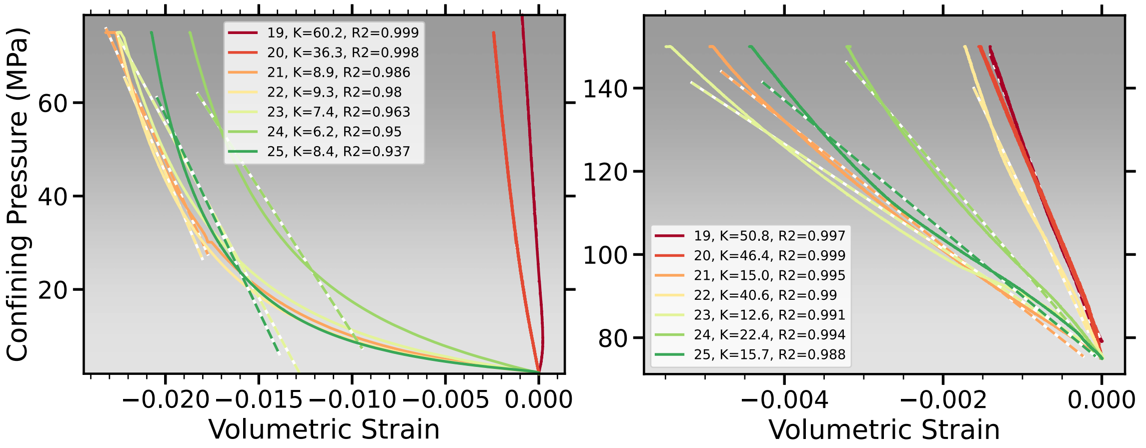

Figure S2: Left: Confining pressure and volumetric strain during hydrostatic loading of samples with (21-26) and without (19 and 20) thermally-induced damage. All samples that were thermally treated show highly non-linear pressure-strain relationships due to the presence of pervasive micro-crack damage, with rapid crack closures at low confining pressures. Right: Same as left panel but after sample fracture and for hydrostatic loading from 75 to 150 MPa. Thermally treated samples exhibit notably lower bulk moduli.

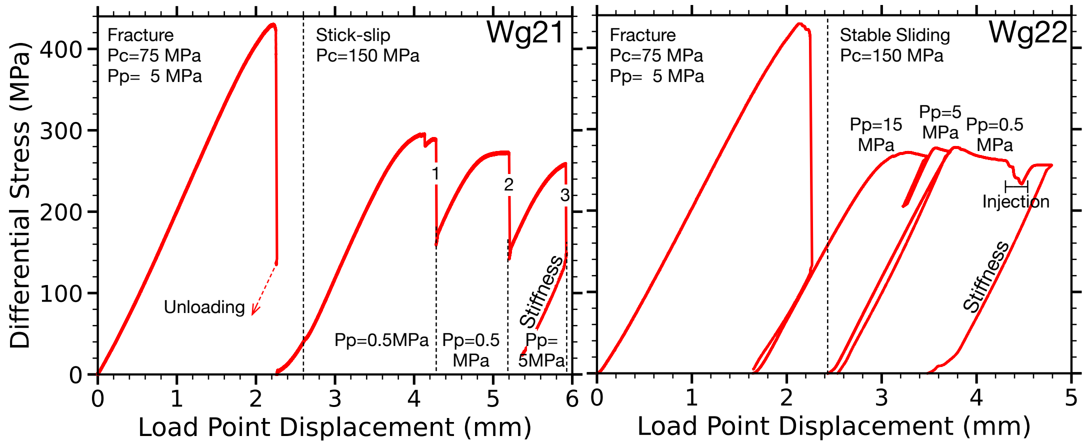

Figure S3: Examples of loading curves for two experiments resulting in stick-slip (left) and stable sliding (right). Initial fracture and frictional sliding periods are separated by vertical dashed lines. Differences in confining and pore pressures are labeled directly for the respective time periods. Experiment 21 resulted in three large stick-slip events (left). Experiment 22 resulted in stable sliding during axial loading at $P_{\mathrm{p}} = 15$ to 0.5 MPa. Subsequent fluid injection at constant axial displacement also resulted in stable stress release. Note that only the first stable sliding phase at $P_{\mathrm{p}} = 15$ MPa is included in the analyses in the main text.

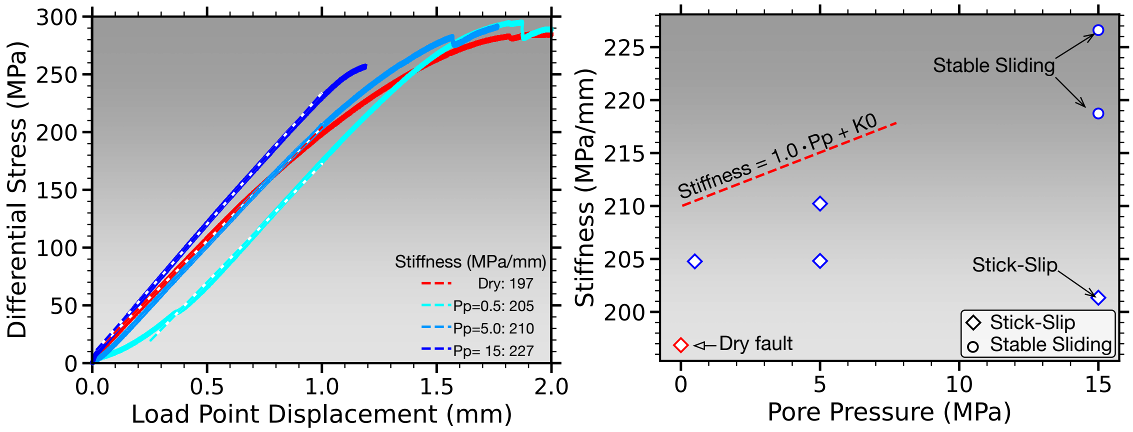

Figure S4: Overview of empirical stiffness estimates (see Method) for the dry experiment (red curve on the left and red diamond on the right) and fluid saturated faults with high-damage and varying pore pressures (blue lines and markers, see legend). High pore pressures and high stiffness always lead to stable sliding, however one test (i.e. 25) at high pore pressures exhibits low stiffness which resulted in stick slip. The red dashed line on the right highlights a potential linear relationship between pore pressure and stiffness.

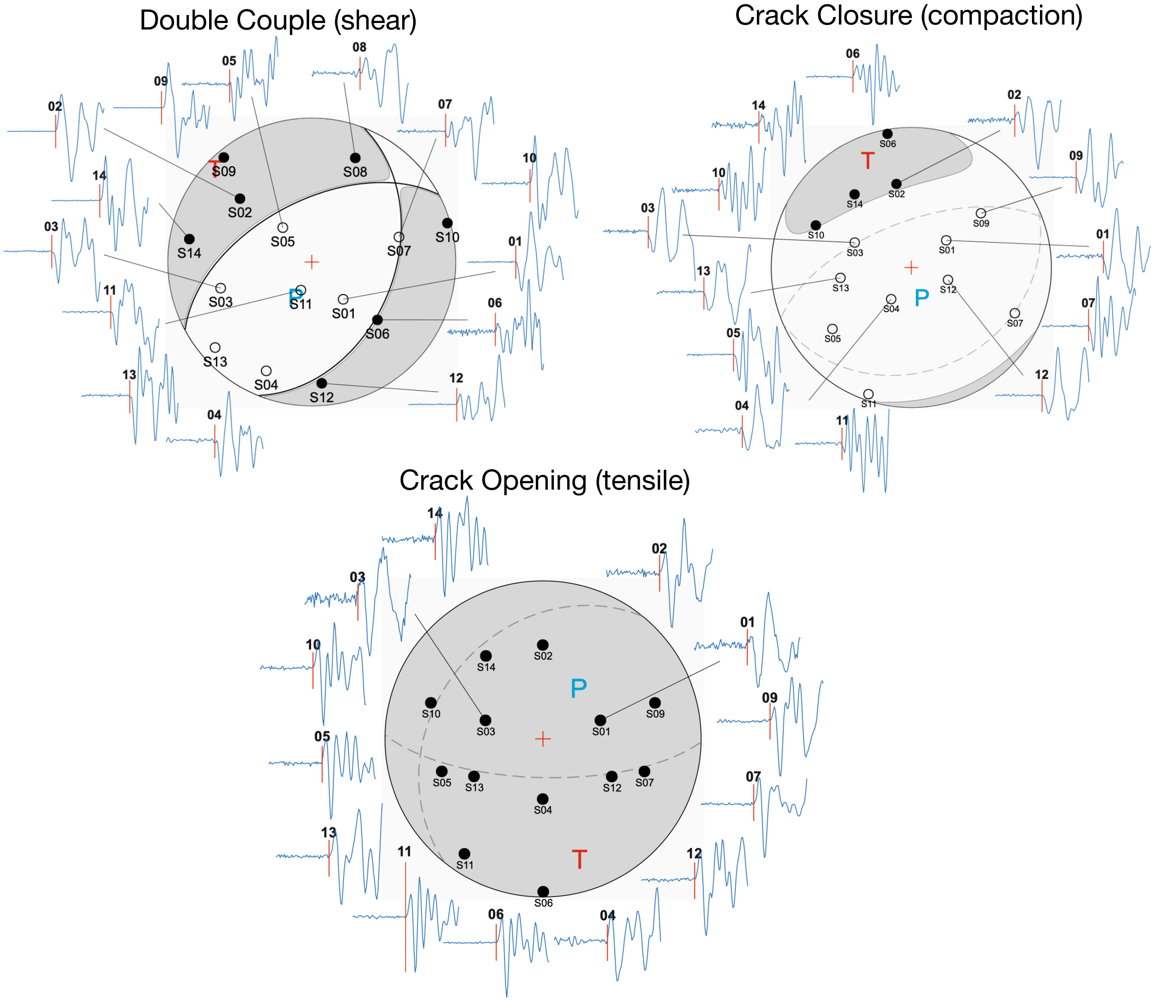

Figure S5: Three exemplary focal mechanisms, take-off angles and normalized first motion amplitudes for a double couple (upper left), crack closure (upper right) and crack opening event (bottom).

Table S2: Macroscopic sample properties (i.e., bulk modulus, B and stiffness, K) during hydro-static and triaxial loading. Note that no strain data was recovered for experiment 26.

ID	B (2-75 MPa) Intact	B (75-150 MPa) Faulted	Stiffness, K
19	60 GPa	50 GPa	197 MPa/mm
20	36 GPa	46 GPa	211 MPa/mm
21	9 GPa	15 GPa	205 MPa/mm
22	9 GPa	40 GPa	219 MPa/mm
23	7 GPa	13 GPa	210 MPa/mm
24	6 GPa	22 GPa	205 MPa/mm
25	8 GPa	16 GPa	201 MPa/mm
26	-	-	227 MPa/mm

S3 Microstructure

We analyzed postmortem micro-structures in micro X-ray computer tomography scan and by optical and scanning electron microscopy (see Method). The appearance of the fault core depends on the used experimental setup and boundary conditions, but is generally 2-3 mm thick and bounded by multiple, undulating principal slip planes inclined at ~30° to the compression axis (Fig. S6). Between the principal slip planes, we observe multiple fractures and thin, subordinate shear planes (<30 μm offset) oriented parallel or 30° to the compression axis, fragmenting the host rock into discontinuous, lenticular pieces. Large, nearly intact host rock pieces with a length of <2 mm were observed in the non-thermally damaged sample (Fig. S6), whereas the fault cores of the thermally damaged samples show the highest fracture density and cataclasis resulting in increased grain size reduction and a wider grain size distribution.

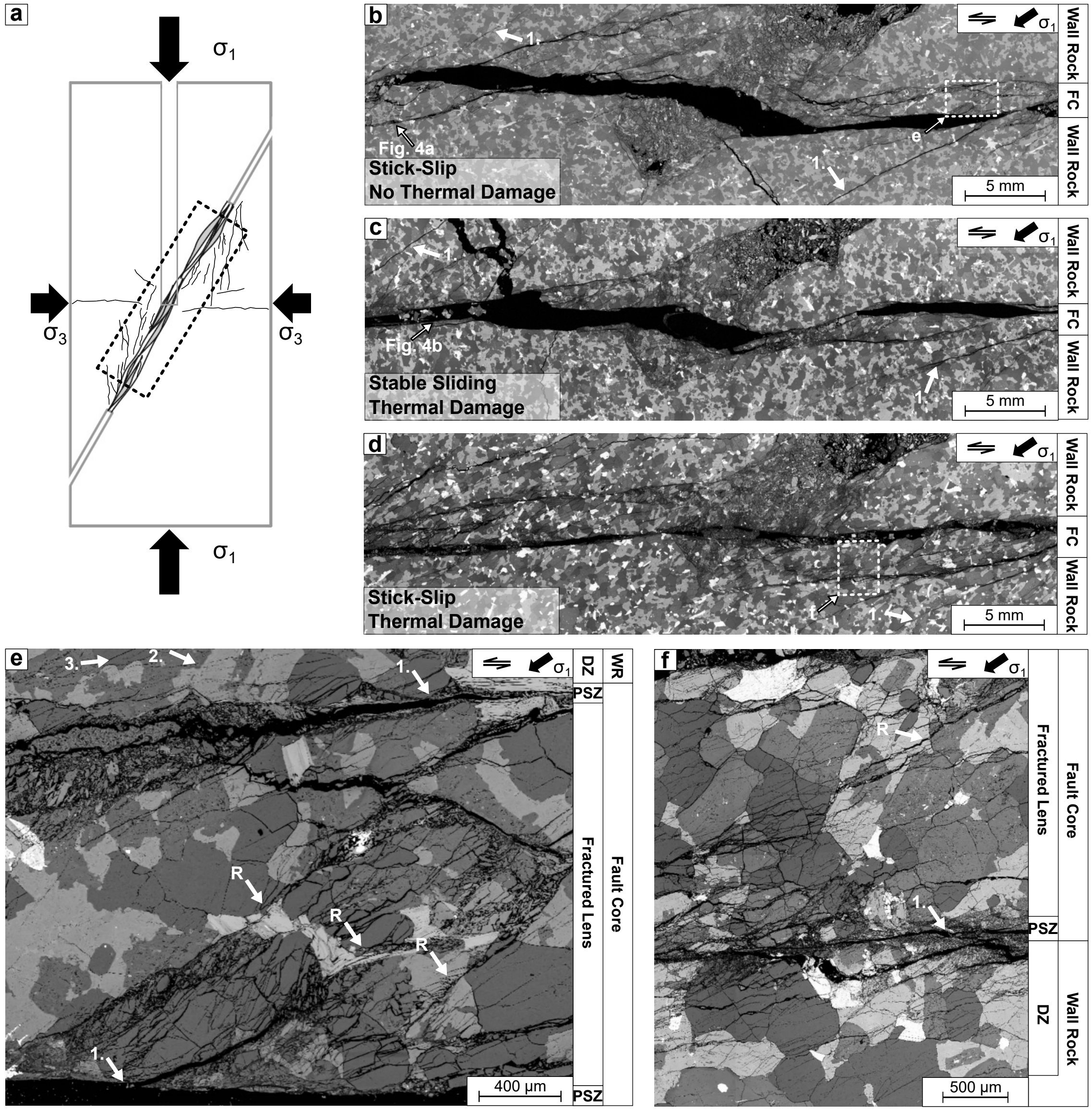

Figure S6: (a) Schematic drawing of average sample geometries, stress orientation and microstructure of all tests. The regions used for micro-analysis of thin sections in b-d are highlighted by the dashed rectangle. (b-d) Microstructures in backscattered scanning electron microscopic images for experiments 20 (b), 22 (c), and 24 (d). White rectangles in b and c indicate the location of the close-up images shown in panels e) and f). White arrows indicate locations of images in Fig. 4 in the main text. (e-f) Fractured lenses within the fault core of the non-thermally damaged sample Panel e) shows a significantly lower fracture density in comparison to the thermally treated sample in f). FC: fault core, WR: wall rock, DZ: damage zone, PSZ: principal slip zone. R: Riedel shears. Numbers are explained in the text.

All samples show the development of an asymmetric damage zone surrounding the fault core (c.f., Fig. S6a), i.e., one side shows relatively few microcracks parallel to the fault zone boundary, while the other side shows a high density of solitary and interconnected trans- and intergranular tensile fractures developed sub-parallel to the main principal stress axis (Fig. S6b, c, d: 1, e:3). Larger interconnected fractures show a slight shear offset, indicated by increased grain size reduction by cataclasis and grain rotation. With decreasing distance to the shear fracture, the microcrack density increases significantly, while crack orientation rotates and becomes predominantly sub-parallel to the shear plane (Fig. S6e: 2). High damage samples exhibit pervasive microcracks throughout the sample (Fig. S7) and higher crack density within the fault damage zone (Fig. S8).

Creeping faults with dominant shear-type events display multiple micrometer-scale shear bands that are aligned parallel (Y-shear) or at an angle of $\sim 20°$ (R1-shear) to the macroscopic shear direction. These samples display the highest density of anastomosing shear planes within the gouge (Fig. 4 c, d, main text). Biotite crystals adjacent to or within the principal slip zones show evidence of crystal plastic deformation (kinking) as well as strong delamination and undulating elongation parallel to the local shear direction. The shear offset of single mica grains along slip zones is variable, suggesting that shear deformation was accommodated by several shear bands at the same time or sequentially with subsequent slip events.

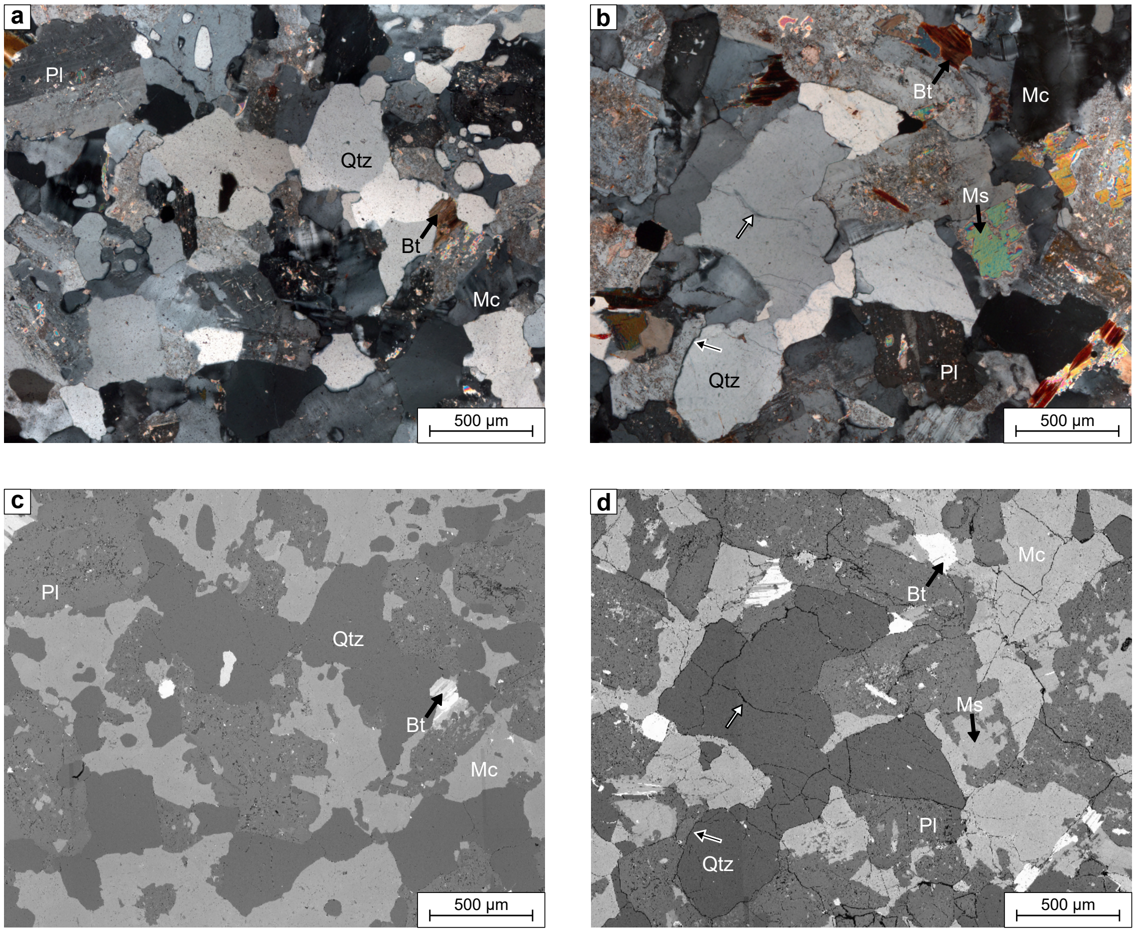

Figure S7: Cross polarized optical (top row) and backscattered electron (bottom row) microscope images of thin sections prepared from the investigated Westerly Granite. In contrast to intact samples (WG20: a, c), thermally treated samples (WG23: b, d) display a high density of thermal stress induced inter- (white bordered arrow) and transgranular (white filled arrow) microcracks of random orientation. Qtz – quartz, Bt – biotite, Pl – plagioclase, Mc – microcline, Ms – muscovite.

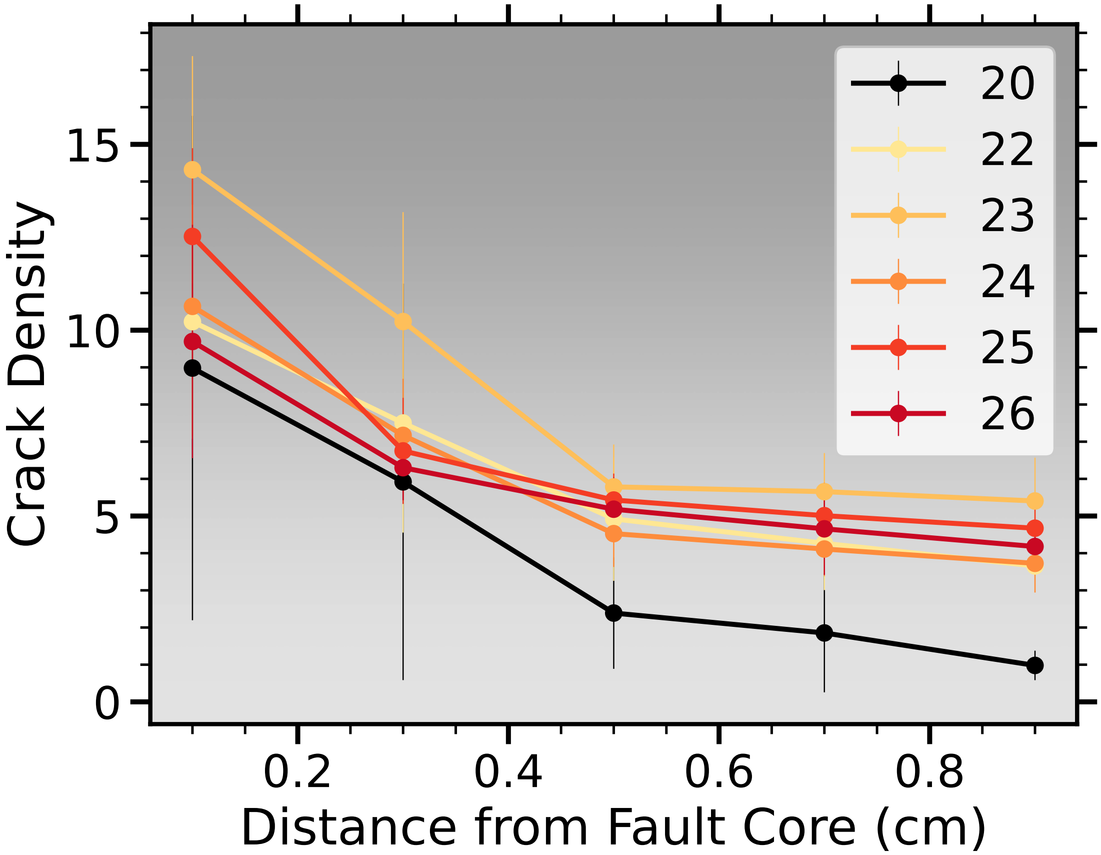

Figure S8: Across fault crack density estimates for experiments 20, and 22 to 26 (see legend). Experiment 20 exhibits notable lower crack densities. All other experiments were thermally treated resulting in relatively higher crack density and fault damage.

S4 Pore volume and velocity change before rock fracture

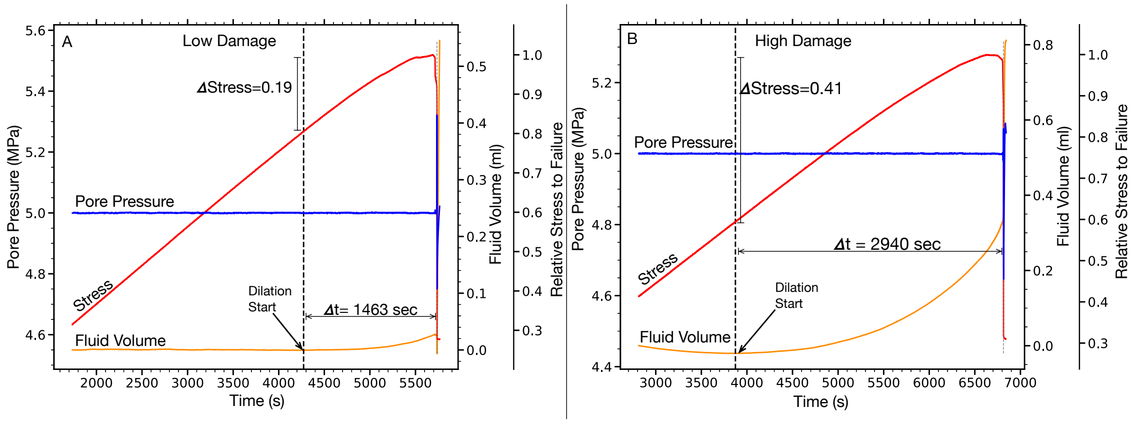

Figure S9: Comparison between pore volume increase before sample fracture at P_c=75 MPa in a low (left) and a high-damage (right) sample. High-initial micro damage leads to about an order of magnitude higher pore volume dilation (orange curves) before fracture. Stresses are highlighted in red and respective stress change and time-to-failure from the pore volume minima are labeled directly on the panels. Pore pressures (blue curve) were servo-controlled at P_p=5 MPa with notable deviations during dynamic fracture.

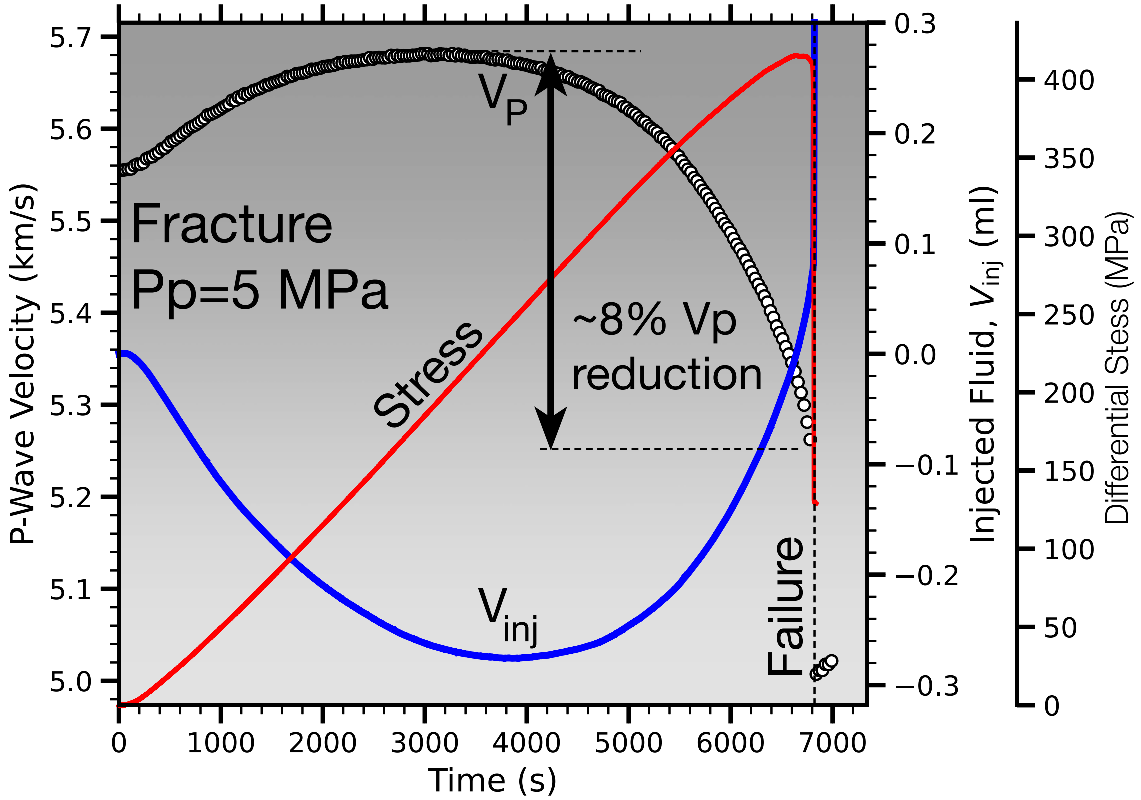

Figure S10: Stress (red curve), pore volume (blue curve) and seismic velocity (black markers) change before dynamic fracture of a high-damage sample. Seismic velocity start to drop substantially before the onset of detectable pore volume increase

S5 Microseismicity rates and focal mechanism variability

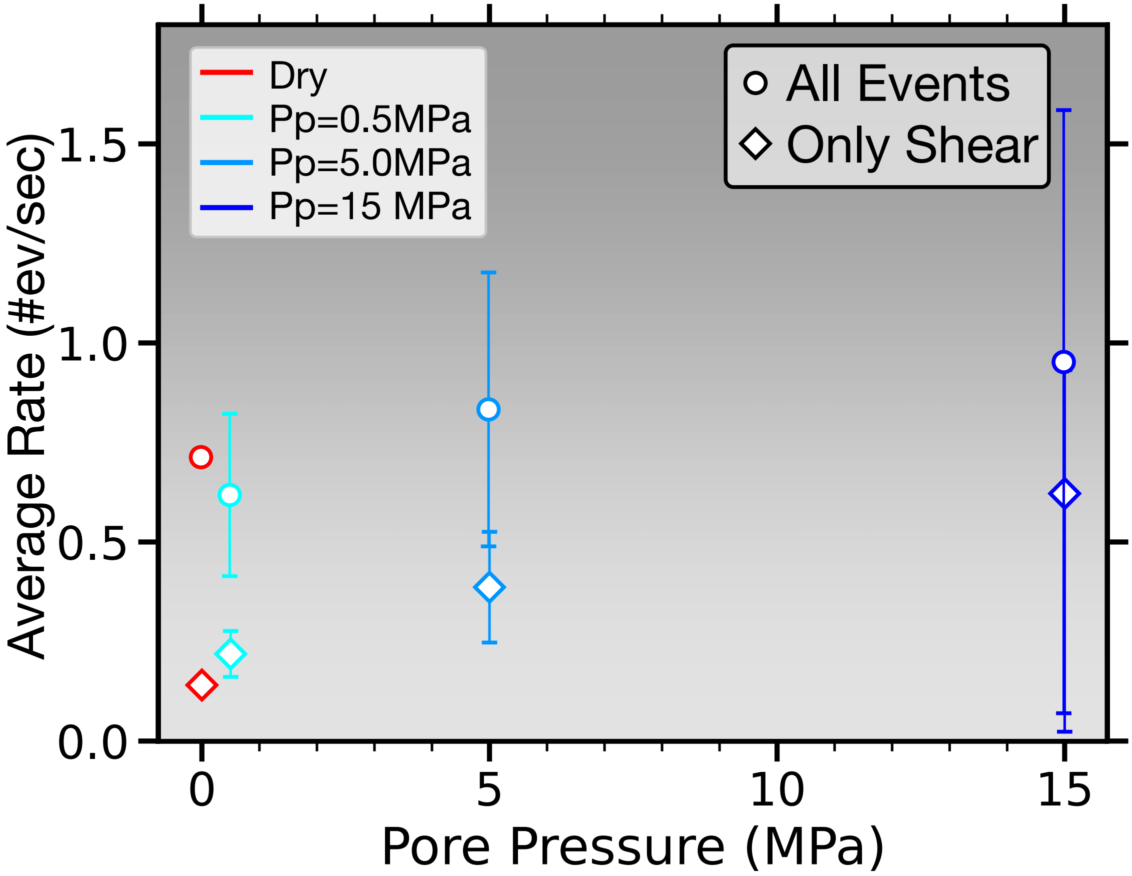

Figure S11: Average AE rates before failure on faults at different pore fluid pressures (see legend). Overall, average rates show a modest increase and high degree of variability for experiments at high pore pressures.

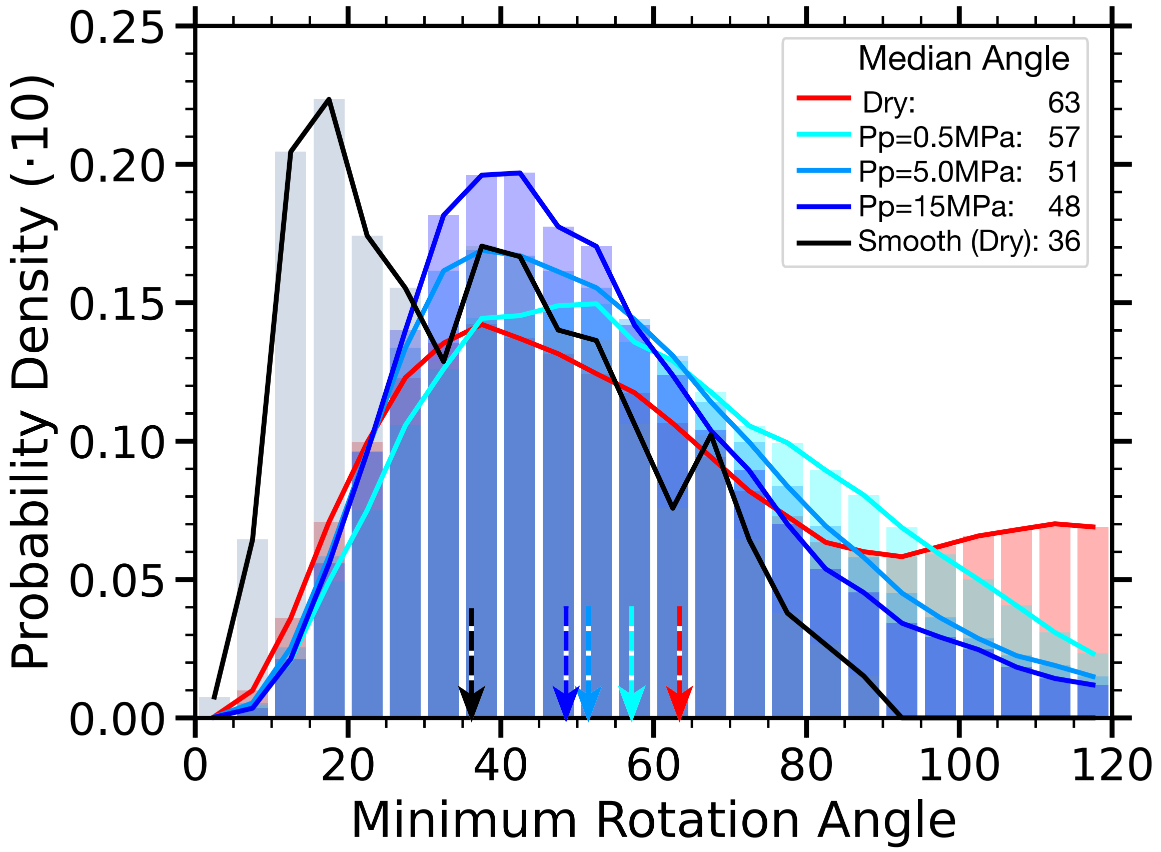

Figure S12: Micro-seismic focal mechanism variability decreases systematically with increasing roughness and fault homogeneity. Focal mechanism variability is determined from the minimum rotation angle required to match the orientation of each pair of AE moment tensors[1,2]. Vertical bars and curves shows the distribution of minimum rotation angles for all possible moment tensor pairs. Colors indicate the following experiments: rough, dry fault (red), P_p=0.5MPa (cyan), light blue (P_p=5MPa), dark blue (P_p=15MPa) and black (smooth, dry fault). The median of each distribution is highlighted by vertical arrows and reported in the legend at the upper right.

References

[1] Tape, W. & Tape, C. Angle between principal axis triples. *Geophysical Journal International* **191**, 813–831 (2012).

[2] Goebel, T. H., Kwiatek, G., Becker, T. W., Brodsky, E. E. & Dresen, G. What allows seismic events to grow big?: Insights from b-value and fault roughness analysis in laboratory stick-slip experiments. *Geology* **45**, 815–818 (2017). URL `http://pubs.geoscienceworld.org/geology/article/45/9/815/208121/What-allows-seismic-events-to-grow-big-Insights`.
